# The Implications of a Dermatopathologist’s Report on Melanoma Diagnosis and Treatment

**DOI:** 10.3390/life13091803

**Published:** 2023-08-24

**Authors:** Asher Nethanel, Christofis Kyprianou, Aviv Barzilai, Ronnie Shapira-Frommer, Yaron Shoham, Rachel Kornhaber, Michelle Cleary, Galit Avinoam-Dar, Shirly Grynberg, Josef Haik, Assaf Debby, Moti Harats

**Affiliations:** 1 Ella Lemelbaum Institute for Immuno-Oncology, Sheba Medical Center, Tel-Hashomer, Ramat Gan 52621, Israel; ronnie.shapira@sheba.health.gov.il (R.S.-F.); shirly.grynberg@sheba.health.gov.il (S.G.); 2 Department of Plastic and Reconstructive Surgery, Sheba Medical Center, Tel-Hashomer, Ramat Gan 52621, Israel; kyprianouchristofis@gmail.com (C.K.); rkornhaber@csu.edu.au (R.K.); dr.avinoamdar@gmail.com (G.A.-D.); josef.haik@sheba.health.gov.il (J.H.); moti.harats@sheba.health.gov.il (M.H.); 3 Department of Dermatology, Institute of Pathology, Sheba Medical Center, Tel-Hashomer, Ramat Gan 52621, Israel; aviv.barzilai@sheba.health.gov.il (A.B.);; 4 Plastic Surgery Department, Burn Unit, Soroka University Medical Center, Faculty of Health Sciences, Ben Gurion University of the Negev, Beer Sheba 84105, Israel; yshoham@bgu.ac.il; 5 School of Nursing, Paramedicine and Healthcare Sciences, Charles Sturt University, Bathurst, NSW 2795, Australia; 6 School of Nursing, Midwifery & Social Sciences, Central Queensland University, Sydney, NSW 2000, Australia; m.cleary@cqu.edu.au; 7 Faculty of Medicine, Tel-Aviv University, Tel-Aviv 69978, Israel; 8 Talpiot Leadership Program, Sheba Medical Center, Tel-Hashomer, Ramat Gan 52621, Israel; 9 Institute for Health Research, University of Notre Dame, Fremantle, WA 6160, Australia

**Keywords:** melanoma, dermatopathology, histopathology review, Breslow’s depth

## Abstract

An accurate and comprehensive histopathology report is essential for cutaneous melanoma management, providing critical information for accurate staging and risk estimation and determining the optimal surgical approach. In many institutions, a review of melanoma biopsy specimens by expert dermatopathologists is considered a necessary step. This study examined these reviews to determine the critical primary histopathology Breslow score in which a histopathology review would be most beneficial. Histopathology reports of patients referred to our institute between January 2011 and September 2019 were compared with our in-house review conducted by an expert dermatopathologist. The review focused on assessing fundamental histologic and clinical prognostic features. A total of 177 specimens underwent histopathology review. Significant changes in the Breslow index were identified in 103 cases (58.2%). Notably, in many of these cases (73.2%), the revised Breslow was higher than the initially reported score. Consequently, the T-stage was modified in 51 lesions (28.8%). Substantial discordance rates were observed in Tis (57%), T1b (59%), T3a (67%) and T4a (50%) classifications. The revised histopathology reports resulted in alterations to the surgical plan in 15.3% of the cases. These findings emphasize the importance of having all routine pathologies of pigmented lesions referred to a dedicated cancer center and reviewed by an experienced dermatopathologist. This recommendation is particularly crucial in instances where the histopathology review can potentially alter the diagnosis and treatment plan, such as in melanoma in situ and thinner melanomas measuring 0.6–2.2 mm in thickness. Our study highlights the significant impact of histopathology reviews in cutaneous melanoma cases. The observed changes in Breslow scores and subsequent modifications in T-stage classification underline the need for thorough evaluation by an expert dermatopathologist, especially in cases of melanoma in situ and thin melanomas. Incorporating such reviews into routine practice within dedicated cancer centers can improve diagnostic accuracy and guide appropriate treatment decisions, ultimately leading to better patient outcomes.

## 1. Introduction

Melanomas represent a small percentage (1.7%) of global cancer diagnoses but account for over 80% of skin cancer deaths [1]. While melanoma is the most lethal form of skin cancer, surgery is often curative when combined with early screening and prevention [2]. Melanoma management poses significant challenges, with plastic surgeons increasingly sought after for their expertise [3]. Therefore, a multidisciplinary approach is required [4], including, but not limited to, surgical oncology, dermatology, plastic surgery, medical oncology, dermatopathology and other health professional specialisms.

Diagnosis of primary cutaneous melanoma relies upon a timely and accurate histopathologic assessment of melanocytic lesions [5]. A precise and comprehensive histopathology report underpins appropriate staging and prognosis, informing the selection of the most suitable surgical treatment [6,7,8,9,10,11,12,13,14,15,16]. The information provided in the histopathology reports is crucial in determining the necessary surgical margins for wide excision and assessing the need for a sentinel lymph node biopsy [17,18].

The pathology report should include all the necessary information required to determine the pathologic T-stage according to the recently published edition of the American Joint Committee on Cancer (AJCC) staging system for melanocytic lesions [19]. The key elements assessed during a histopathologic examination include the Breslow thickness, the ulceration status, and the presence or absence of microsatellites, all of which are essential prognostic factors. Additional prognostic criteria such as the deep and peripheral margins status, the mitotic rate, presence of regression, lympho-vascular invasion, tumor-infiltrating lymphocytes and neurotropism are also important characteristics that help to decipher the tumor’s behavior [19,20]. These factors contribute to accurate staging and provide important prognostic information which helps determine the risk of disease progression and metastasis. The Breslow thickness specifically influences the decision-making process for surgical treatment, with thicker tumors often requiring wider excision margins to ensure complete tumor removal. Furthermore, the histopathology report assists in determining whether a sentinel lymph node biopsy is warranted. This procedure plays a crucial role in assessing regional lymph node involvement and aids in the identification of patients who may benefit from further adjuvant therapy [21,22].

Recognizing the significance of accurate histopathologic assessment in primary cutaneous melanoma, various melanoma guidelines and workgroups emphasize the importance of consistent and standardized reporting of pathology information. These guidelines recommend a synoptic reporting approach, which entails the systematic documentation of all relevant parameters discussed above. Synoptic reporting ensures that essential histopathologic features are consistently and comprehensively included in the pathology report. This structured approach helps streamline communication between pathologists and clinicians [15,16,18,23]. Furthermore, the guidelines highlight the significance of having pathologists with expertise in evaluating pigmented lesions to perform the histopathologic assessment of melanoma specimens [24].

The definitive diagnosis of invasive melanoma versus dysplastic non-invasive melanocytic lesions can be challenging. Pathologists experienced in this specialized field possess in-depth knowledge of the morphologic characteristics of melanocytic lesions and are familiar with the subtle nuances that differentiate benign lesions from malignant ones. Their expertise allows for accurate measurement and interpretation and precise reporting of an accurate T-stage for invasive melanomas [6,8,9,10,11,12,13,14,15,25,26,27,28,29,30]. An inaccurate diagnosis may lead to inadequate estimation of the recurrence risk and thus to over- or under-treatment [6,10,12,13,15,16,29]. Therefore, a review of melanoma biopsy specimens by an expert dermatopathologist in pigmented lesions is a common practice in several tertiary care referral centers in the world, in an effort to reduce the risk of misdiagnosis, reduce the deviation of staging and to facilitate the optimal management of patients diagnosed with primary cutaneous melanoma [6,10,12,15,16,29,31,32,33].

Despite the presence of practical guidelines for histopathology reviews in cutaneous melanoma, a disparity frequently arises between the level of effort dedicated to the reviews and the tangible outcomes they yield. This discrepancy raises important considerations, including the personnel effort and economic burden associated with conducting internal histopathology reviews. Additionally, the potential delay in therapeutic decision making due to the review process further highlights the need to assess the extent and impact of the differences between the initial pathology reports and the revised ones.

To address these concerns, our study aimed to determine the critical primary pathology Breslow score at which a histopathology review would be most beneficial. By investigating the discrepancies between initial and revised pathology reports, we sought to examine the value and potential impact of conducting histopathology reviews. Identifying the specific Breslow score at which the revisions were most prevalent and impactful would allow for a more targeted allocation of resources by ensuring that the effort and cost associated with conducting internal reviews are justified, would help minimize the delay in therapeutic decision making and, finally, would provide valuable insights into the overall accuracy and reliability of the initial diagnoses. This information can guide quality improvement initiatives and enhance the overall diagnostic process in cutaneous melanoma.

## 2. Materials and Methods

### 2.1. Ethics Statement

This study was approved by the Helsinki Committee at the Sheba Medical Center, Tel Hashomer, Israel (SMC–17-4160).

### 2.2. Patients

Data were derived from our melanoma registry—a prospectively updated, medical-record-based registry. Eligible patients were diagnosed with cutaneous melanoma according to an external histopathology report, without clinical evidence of nodal invasion or distant metastases, and were referred to our institute for surgical and oncological consultation between January 2011 and September 2019. Patients diagnosed with malignant melanoma by an in-house histopathology report, patients with mucosal melanoma and pediatric patients were excluded from the study.

We collected demographic, clinical, and histopathological data. Details regarding the primary melanoma histopathology were obtained from pathological reports and included the type of biopsy conducted and all available histopathologic prognostic features reported by the pathologist. Surgical data from external histopathology reports and in-house reviews included the margins of resections and details on wide local excision and the sentinel lymph node biopsy.

### 2.3. Histopathology Reviews

Patients referred to the Department of Plastic and Reconstructive Surgery, Sheba Medical Center, Israel, for surgical intervention for cutaneous malignant melanoma with a confirmed diagnosis are required to bring their external histopathology slides and reports for a second opinion. This institutional policy requires that all suspected cutaneous malignancies be evaluated by a single certified in-house dermatopathologist.

In this study, we compared the external reports with the institutional review, looking at cardinal prognostic histological features: the Breslow thickness and ulceration. Further analysis was undertaken for available cases to compare the mitotic rate, Clark level, vascular invasion, neurotropism, tumor-infiltrating lymphocytes, excision margins and regression. The dermatopathologists did not have access to the external histopathology report when performing their reviews.

### 2.4. Statistical Analysis

Descriptive statistics were used to describe patient characteristics and clinical as well as histopathological features of their melanoma lesions. For quantitative variables, we utilized mean values and their relative standard deviations. For nominal variables, we utilized frequencies. Differences among quantitative variables were evaluated using the parametric T-test, and the chi-square test was used to evaluate differences among categorical variables. Statistical significance was defined at the *p* ≤ 0.05 level, and all tests were two-sided.

## 3. Results

### 3.1. Population Characteristics

We identified 321 consecutive melanoma patients who underwent surgery in the study setting. Of these, histopathology reviews by a qualified dermatopathologist were conducted on 177 specimens (55.14%). Two patients were identified with two simultaneous primary malignant melanomas. The patients’ mean age was 60.6 years ± 14.7 (range 25–86). Ninety (51.4%) of the patients were female and eighty-five (48.6%) were male. A total of 11 patients (6.3%) had a personal history of melanoma, whereas 164 (93.7%) did not. Five patients (2.8%) had a first-degree family history of melanoma. Lesions were most often located on the torso (*n* = 79, 44.6%), followed by the lower extremities (*n* = 42, 23.7%), upper extremities (*n* = 37, 20.9%) and the head and neck (*n* = 19, 10.7%). Nearly half of the lesions (*n* = 85, 48.3%) were in sun-exposed areas. Most of the primary excisions that were performed were excisional biopsies (*n* = 158, 89.3%), followed by incisional biopsies for wider lesions (*n* = 11, 6.2%), shave biopsies (*n* = 6, 3.4%) and Mohs surgery biopsies (*n* = 2, 1.1%). No data were available as to the reason for shave or Mohs biopsies, as those were conducted in the community.

The mean Breslow index was 1.6 ± 1.6 mm and the median was 1.0 mm (range 0–8.0 mm). Ulceration was documented in 23 lesions (13%). Ulcerated lesions had a higher Breslow index compared to non-ulcerated lesions (mean 2.8 mm ± 0.4 vs. 1.5 mm ± 0.1, *p* = 0.0001). Margins were involved in the primary biopsy in 33 cases (18.6%), of which 11 were expected (Mohs, punch, and shave biopsies).

Wide local excision was performed in 173 patients for 175 specimens. The clinical excision margins performed were 2 cm in 88 cases (50.3%), 1.5 cm in 6 cases (3.4%), 1 cm in 67 cases (38.3%) and 0.5 cm in 14 cases (8%). Residual malignancy was reported in the histopathologic report of the wide local excision in 28 cases (16%); however, in 17 (60.7%) of these cases, despite the presence of residual malignancy, the excisional biopsies exhibited uninvolved margins and met the necessary guideline properties for adequate surgical margins.

A sentinel lymph node biopsy was performed in 119 cases (67.2%), of which 15 (12.6%) had a positive sentinel node. As expected, those with a positive sentinel node had a higher rate of ulcerated primary melanoma (*p* = 0.03) and had a significantly higher Breslow index (mean 3.4 mm vs. 1.9 mm, *p* = 0.0005). Patients’ characteristics are summarized in Table 1.

### 3.2. Prognostic Implications of Histopathology Reviews

**Figure 1:**
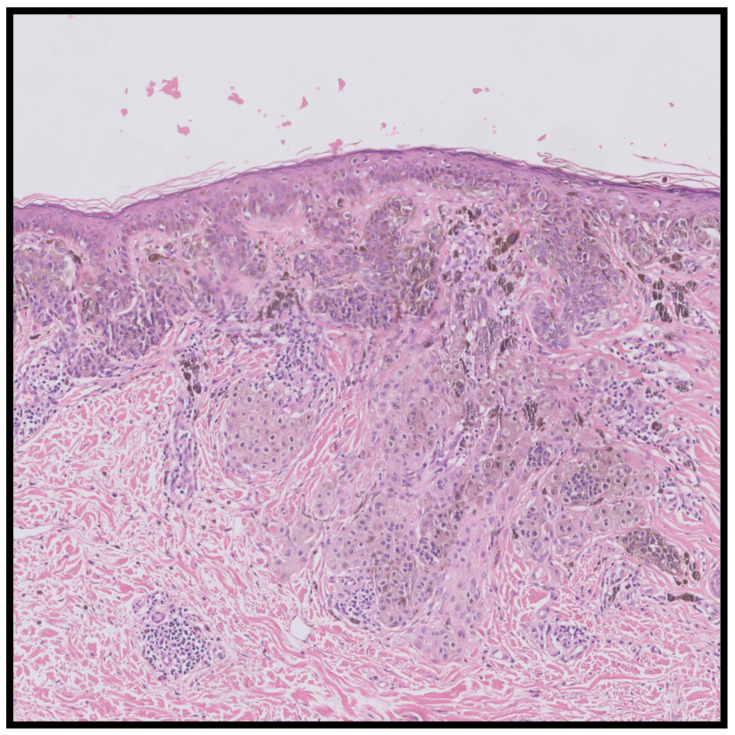
This case was downgraded from an invasive malignant melanoma, Breslow thickness of 0.6 mm to a dysplastic nevus with only the possibility of an evolving malignant melanoma in situ. The histology shows junctional atypical nests and single melanocytes with fusion of the rete ridges and some papillary dermal fibrosis. The dermal component is nested and composed of more bland melanocytes.

**Figure 2:**
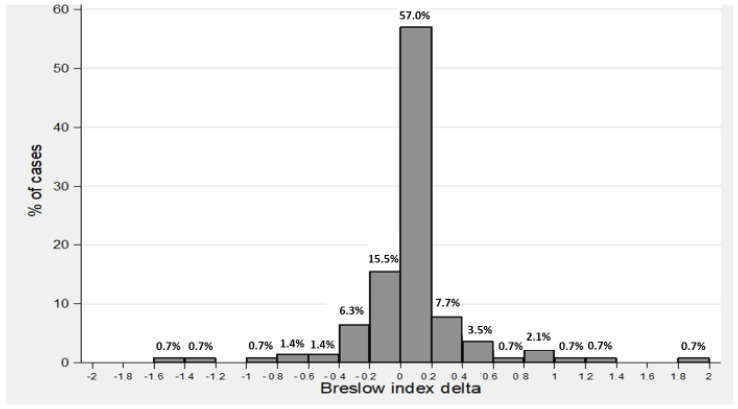
Distribution of Breslow deltas.

As a result of the histopathology reviews, the Breslow index was changed in 103 cases (58.2%). In most of the cases (72.5%), the change in Breslow was less or equal to 0.2 mm and in the other 27.5% of the cases, the difference between the initial and the revised Breslow was higher than 0.2 mm (range −6.0 mm to +2.0 mm). In nearly three-quarters of the cases (73.2%), the review detected a higher Breslow index than initially reported, and in 26.8%, the review downgraded the Breslow index (see Figure 1). Figure 2 describes the distribution of Breslow indexes pre- and post-review and their relative deltas.

To evaluate the effect of the baseline patient or initial lesion characteristics on the extent of Breslow index changes on histopathology review, we performed a regression analysis with selected baseline characteristics. We found no significant correlation with the age nor the gender of the patient, and no correlation with the site of the lesion nor with whether the lesion was biopsied from a sun-exposed area or not. The type of biopsy was also not correlated with delta. After exclusion of extremity cases, we found that the initial Breslow index, as reported in the initial analysis, was also not correlated with delta.

While investigating other revised histopathologic features, we found the ulceration status was changed in 22 of the 168 histopathology reports that contained ulceration status (13.1%) and the mitotic rate was changed in 47 of the 91 histopathology reports that contained mitotic rate data (51.6%). Other changes included a 21.4% change in Clark level, a 12.5% change in the presence of tumor infiltrating leukocytes, a 5.4% change in the status of the excision margins and a 5% change in signs of regression. The detailed changes in all histopathologic features are summarized in Table 2.

As a consequence of the revised histopathologic features, the T stage was changed in 51 lesions (28.81%). Table 3 specifies the change in T stages after review. Low concordance rates between external and internal histopathology reports were seen in Tis (57%) and in T1b (59%). In both cases, the review upgraded or downgraded the T-stage to a similar extent. Low concordance rates were also seen in T3a (67%) and in T4a (50%). In these higher T-stages, all revised pathologies upgraded the staging.

In addition to the changes in prognosis T-stage subgroups, there were three cases of misdiagnosis regarding the presence of malignancy in the specimen. Specifically, one case of an initially diagnosed benign nevus was changed to in situ melanoma and two cases of initially diagnosed in situ melanomas were changed to benign nevi.

### 3.3. Surgical Implication of Histopathology Reviews

As described in Table 4, the revised histopathology report with the aforementioned histopathologic changes led to changes in the surgical plan in 15.3% of the cases (27 of the 177 reviews). In 13 cases (7.3%), surgical plans were changed regarding the need for a sentinel lymph node biopsy. In six cases, the revised histopathology led to a decision to perform a sentinel lymph node biopsy, whereas in seven cases, it led to the omission of this procedure. Decisions to change the extent of surgical margins were taken in 17 patients (9.6%). Fourteen patients underwent larger margin excisions than initially planned for and three underwent smaller margin excisions.

## 4. Discussion

Imprecise or incomplete histopathologic reports of melanocytic lesions can have far-reaching consequences, potentially leading to inappropriate treatment recommendations and misguided decisions regarding follow-up care for melanoma patients. Beyond the obvious medico-legal implications, the consequences of such errors in surgical treatment can significantly compromise patient outcomes [6,10,12,15,16,17,26,29].

The issue of inter-observer variations and disagreements among pathologists regarding the diagnosis and staging of suspected melanocytic lesions has been widely recognized and extensively studied. Previous research has reported discordance rates ranging from 9% to 24% [6,8,11,12,13,15,16,17,27,33]. This highlights the considerable challenge in achieving consistent and reliable histopathologic assessments in melanoma cases. To address this challenge, it is imperative that all biopsies of suspected pigmented lesions undergo a comprehensive review by experienced dermatopathologists who specialize in melanocytic lesions [31,32,33]. The involvement of experienced dermatopathologists in the review process plays a crucial role in minimizing inter-observer variability and ensuring accurate diagnoses. These specialized pathologists possess extensive knowledge and expertise in the evaluation of melanocytic lesions, enabling them to discern subtle morphologic differences and distinguish between benign and malignant lesions with greater accuracy. Their thorough understanding of the diagnostic criteria and prognostic indicators for melanoma enhances the reliability of histopathologic reports and facilitates appropriate treatment decision making.

In our study, we reported on 177 consecutive cases that were routinely reviewed by our in-house dermatopathologist. Most of the lesions (44.6%) were removed from the torso and only 10.7% from the head and neck area. The median Breslow index was 1.0 mm, ranging from 0 mm (no invasion) to 8.0 mm. A sentinel lymph node procedure was completed in 67.2% of the cases, and 12.6% were positive. As expected, there was a significant correlation between the Breslow index and the presence of ulceration, as well as with positivity of sentinel lymph node biopsy.

After review, the diagnosis of melanoma was changed in three cases (1.7%), where one case of benign nevus was changed to in situ melanoma and two cases of in situ melanomas were changed to benign nevi. This number is below the range reported in other studies (1.2–14%) [27,33], where dysplastic nevi were the most important source of false positive diagnoses, mainly in situ melanomas [8]. The Breslow index was changed in 58.2% of the cases after review by our dermatopathologists. In most of the cases (72.5%), the corrected Breslow was within a 0.2 mm range from the original value, yet in 73.2%, the new Breslow index was upgraded (higher number). In 13.1% of the cases, a change in ulceration status was also registered. Other studies report smaller numbers [8], with ulceration as the most reproducible histologic feature [9,16,28,30]. The mitotic rate was changed in 51.6% of the reviewed specimens. The mitotic rate is an important prognostic factor that was used for the AJCC classification of T1 melanomas in the 7th edition [34]. According to the 8th edition, T1 is now sub-categorized to T1a and T1b according to Breslow thickness with a 0.8 mm stratum, which is a more powerful prognostic factor compared to the mitotic rate as a dichotomous variable [19,20].

As a result of the Breslow and ulceration status changes, the prognostic T-stage classification was changed in 28.8% of the cases. Low concordance rates were seen in thin melanomas, namely melanoma in situ (57%) and in T1b (59%) in this study, where the review resulted in upgrading or downgrading the T-stage to the same extent. This was also reported in other research from Australia and the US [6,14]. The accuracy in the diagnosis of both melanoma in situ and T1b melanoma is crucial for treatment planning, since both are decision-changing points that affect the extent of excisional margins (0.5 cm for melanoma in situ vs. 1 cm for invasive thin melanoma) and the decision to perform a sentinel lymph node biopsy or not (not recommended in T1a vs. offered in T1b). Concordance was also relatively low for T3a (67%) and T4a (50%), where all the revised pathologies were upstaged. This affects mostly the prognosis and the follow up, but in certain circumstances may impede inclusion in adjuvant trials for high-risk stage II melanoma [35].

Of the 177 reviews, the changes in T-stage caused a change in the surgical plan in 15.3% of the cases. In 9.6% of the cases, the decision on the extent of margin excision was changed (mostly enlarged as a result of the Breslow index upgrade), and in 7.3%, the decision on sentinel lymph node biopsy was reversed—whether omitted or performed—to the same extent. Contrary to other studies [6,33], we did not find any association between the extent of change in Breslow index and the patients’ or tumors’ baseline characteristics, specifically neither the site or type of biopsy nor the age or sex of the patient.

Our study has several strengths and limitations. The study analyzed a substantial sample size of specimens, providing a comprehensive evaluation of the impact of histopathology reviews on cutaneous melanoma diagnosis and treatment planning and enhancing the reliability of the findings. Furthermore, this study was conducted in a tertiary reference medical center, which specializes in the treatment of melanoma, and the review was performed by an experienced dermatopathologist, ensuring a high level of expertise and accuracy in the assessment of histologic and clinical prognostic features. The involvement of an expert adds credibility to the revised Breslow scores and subsequent changes in T-stage classification. The authors of this study acknowledge the fact that a pathology revision might potentially cause a delay to the surgical plan if not conducted in a timely manner. In our institute, pathological revisions were generally completed within 10–14 days, yet the surgical intervention was planned and scheduled while awaiting the results of the review to avoid unnecessary delays. Other limitations of this study are its retrospective nature and the fact that this study was conducted at a single institute, which may limit the generalizability of the results to other settings or populations. Variations in diagnostic practices and expertise across different institutions may affect the extent to which histopathology reviews impact diagnosis and treatment planning.

## 5. Conclusions

Despite the reported improvements in inter-observer concordance and reproducibility following the implementation of the 8th edition of the AJCC classification, variability in histopathologic assessments of cutaneous melanoma remains a significant concern [36]. This variability directly impacts the accurate estimation of prognosis and the selection of the most suitable surgical plan for patients. Based on our findings, we strongly recommend that all routine histopathology of pigmented lesions referred to a dedicated cancer center undergo thorough review by an experienced dermatopathologist. By mandating comprehensive reviews by experienced dermatopathologists, healthcare institutions can mitigate the risks associated with inter-observer variations and ensure that patients receive accurate diagnoses and appropriate treatment recommendations. Furthermore, ongoing quality assurance initiatives, including regular feedback and educational programs for pathologists, can contribute to reducing the discordance rates and improving the overall consistency of histopathologic assessments.

A mandated histopathology review is particularly crucial in decision-changing instances, such as melanoma in situ and thin melanomas measuring 0.6–2.2 mm, where even a small 0.2 mm change in the Breslow index could significantly alter the surgical plan. By ensuring the involvement of experienced dermatopathologists and considering minimal threshold changes, we can enhance the precision and reliability of histopathologic assessments, leading to improved patient outcomes, optimized treatment planning and a more accurate prognostic estimation in cutaneous melanoma management. Continued efforts to address the remaining variability in histopathologic assessments are essential for enhancing the overall quality and consistency of care provided to patients with cutaneous melanoma.

## Figures and Tables

**Table 1:** Patients’ characteristics.

Total Patients		*n* = 175
Specimens		*n* = 177 *
Sex	Male	85 (48.6%)
	Female	90 (51.4%)
Age (mean ± SD)		60.6 ± 14.7
Personal history of melanoma	Yes	11 (6.3%)
	No	164 (93.7%)
Family history	Yes	5 (2.9%)
	No	170 (97.1%)
Lesion location	Head and Neck	19 (10.7%)
	Torso	79 (44.6%)
	Upper Extremities	37 (20.9%)
	Lower Extremities	42 (23.7%)
Sun-exposed area	Yes	85 (48.3%)
	No	91 (51.7%)
Type of biopsy	Excisional biopsy	158 (89.3%)
	Incisional Biopsy	11 (6.2%)
	Shave biopsy	6 (3.4%)
	Mohs	2 (1.1%)
Surgical treatment after the histopathology review	Wide excision	57 (32.8%)
	Wide excision + SLNB	119 (67.2%)

SD: standard deviation, SLNB: sentinel lymph node biopsy. * Two patients were identified with two simultaneous primary malignant melanomas.

**Table 2:** Histopathology review changes.

Features	N Change	% Change
Breslow	103/177	58.2%
Ulceration	22/168	13.1%
Mitotic Rate	47/91	51.6%
Clark Level	6/28	21.4%
Vascular Invasion	0/10	0%
Neurotropism	0/6	0%
Tumor-Infiltrating Leucocytes	1/8	12.5%
Regression	1/20	5%
Margin Status	7/129	5.4%

**Table 3:** Changes in diagnosis and staging between initial external histopathology reports and internal revised histopathology review.

Initial T-Stage	Revised T-Stage										
	Benign	Tis	T1a	T1b	T2a	T2b	T3a	T3b	T4a	T4b	Total
Benign	0	1	0	0	0	0	0	0	0	0	**1**
Tis	2	8 (57%)	3	0	0	1	0	0	0	0	**14**
T1a	0	0	35 (80%)	8	1	0	0	0	0	0	**44**
T1b	0	0	8	22 (59%)	7	0	0	0	0	0	**37**
T2a	0	1	0	2	19 (79%)	2	0	0	0	0	**24**
T2b	0	0	0	0	2	11 (85%)	0	0	0	0	**13**
T3a	0	0	0	0	0	0	12 (67%)	5	0	1	**18**
T3b	0	0	0	0	0	0	0	8 (100%)	0	0	**8**
T4a	0	0	0	0	0	0	0	0	5 (50%)	5	**10**
T4b	0	0	0	0	0	0	0	0	2	6 (75%)	**8**
Total	**2**	**10**	**46**	**32**	**29**	**14**	**12**	**13**	**7**	**12**	**177**

Tis: T stage in situ.

**Table 4:** Implications of the histopathology review on the surgical plan.

Consequential Change	*n* (%)
Change to AJCC T-Stage.	51 (28.8%)
Change to surgical plan	27 (15.3%)
Surgical Margins	17 (9.6%)
enlarged	14 (7.9%)
reduced	3 (1.7%)
SLNB requirement	13 (7.3%)
undertaken	6 (3.4%)
omission	7 (3.9%)

AJCC: American Joint Committee of Cancer Staging System, SLNB: sentinel lymph node biopsy.

## Data Availability

The data can be requested from the authors.
